# Quantum Channel Extreme Bandgap AlGaN HEMT

**DOI:** 10.3390/mi15111384

**Published:** 2024-11-15

**Authors:** Michael Shur, Grigory Simin, Kamal Hussain, Abdullah Mamun, M. V. S. Chandrashekhar, Asif Khan

**Affiliations:** 1 Department of Electrical, Computer, and Systems Engineering, Rensselaer Polytechnic Institute, Troy, NY 12180, USA; 2 Department of Electrical Engineering, University of South Carolina, Columbia, SC 29208, USA; simin@engr.sc.edu (G.S.); mm88@email.sc.edu (A.M.); chandra@cec.sc.edu (M.V.S.C.); asif@engr.sc.edu (A.K.); 3 Department of Chemistry and Biochemistry, University of South Carolina, Columbia, SC 29208, USA; mhussain@email.sc.edu

**Keywords:** HEMT, AlGaN, quantization, breakdown field, mobility

## Abstract

An extreme bandgap Al_0.64_Ga_0.36_N quantum channel HEMT with Al_0.87_Ga_0.13_N top and back barriers, grown by MOCVD on a bulk AlN substrate, demonstrated a critical breakdown field of 11.37 MV/cm—higher than the 9.8 MV/cm expected for the channel’s Al_0.64_Ga_0.36_N material. We show that the fraction of this increase is due to the quantization of the 2D electron gas. The polarization field maintains electron quantization in the quantum channel even at low sheet densities, in contrast to conventional HEMT designs. An additional increase in the breakdown field is due to quantum-enabled real space transfer of energetic electrons into high-Al barrier layers in high electric fields. These results show the advantages of the quantum channel design for achieving record-high breakdown voltages and allowing for superior power HEMT devices.

## 1. Introduction

**Figure 1:**
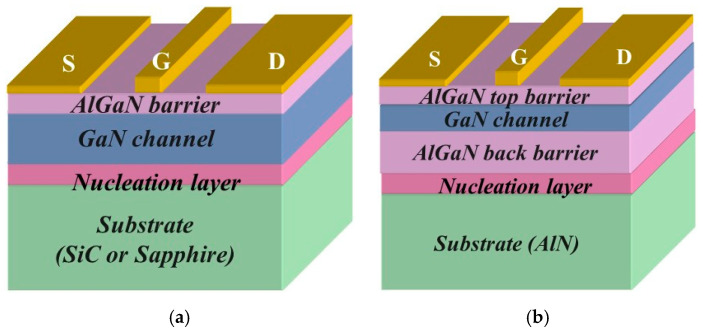
Conventional (**a**) and QC-HEMT (**b**) designs. QC-HEMT in (**b**) can have GaN or AlGaN channel (GaN channel is shown).

Ever since their emergence in the 1990s and early 2000s [1,2,3,4,5,6] GaN high-electron-mobility transistors have demonstrated superior performance for high-voltage [7,8,9,10], high-frequency [11,12,13,14], and high-temperature [12,15] operation, as well as excellent radiation hardness [16,17]. The primary reasons for their superiority for operation in extreme environments are a wide energy gap, leading to high breakdown voltage, and polarization doping [18,19,20,21], which supports large sheet electron densities in the HEMT channels. The high polar optical phonon energy in GaN (91.2 meV [22,23,24] compared to 35 meV for GaAs [25,26,27]) and the screening of impurity scattering by a large electron density in the device channel ensure high field-effect mobility, especially in the on-state, and lead to low on-resistance for long [28,29,30,31,32,33,34,35,36] and short channel devices [37]. In this paper, we show that these advantages could be dramatically enhanced using the Quantum Channel HEMT (QC-HEMT) design. QC-HEMT incorporates a thin channel comparable sandwiched between the top and bottom wide bandgap barriers. Figure 1 compares conventional HEMT and QC-HEMT designs.

In contrast to conventional GaN HEMT designs, the shape of the potential confining two-dimensional electron gas (2DEG) in QC-HEMT is determined by both the polarization field and bandgap discontinuities at the top and bottom barrier interface. Carrier confinement in the HEMT 2D electron gas (2DEG) channel affects most device performance parameters. Strong electron confinement reduces trapping and related gate and drain lags, increases channel mobility, and helps achieve lower contact resistance. However, in conventional single heterojunction HEMTs, band diagrams flatten out at gate voltages near or below the threshold, reducing or eliminating electron confinement. As we show in this work, in addition to maintaining confinement, the quantization of 2DEG in the QC design enables the effect that we call quantum real space transfer (QRST). QRST leads to achieving breakdown fields exceeding those predicted by the material properties of the device channel.

The QC-HEMT advantages could be greatly enhanced using extreme bandgap (EBG) semiconductors with bandgap energy exceeding 4–5 eV, such as AlGaN with Al-fraction x > 0.6. These devices show strong promise for achieving record-high breakdown voltage, high-temperature operation, radiation hardness, and chemical stability [38,39,40,41,42,43,44,45,46].

For high-power III-Nitride EBG devices, the choice of substrate is of critical importance. Substrate lattice matching to device active layers is needed to avoid strain relaxation and defect generation. Bulk AlN is thus the best choice of substrate for high-Al AlGaN devices [47,48,49,50,51,52] and we employ it in our design. Bulk AlN substrate also has high thermal conductivity, providing efficient thermal management of high-power devices.

## 2. QC-HEMT Design and Key Properties

### Band Diagrams and Energy States

**Figure 2:**
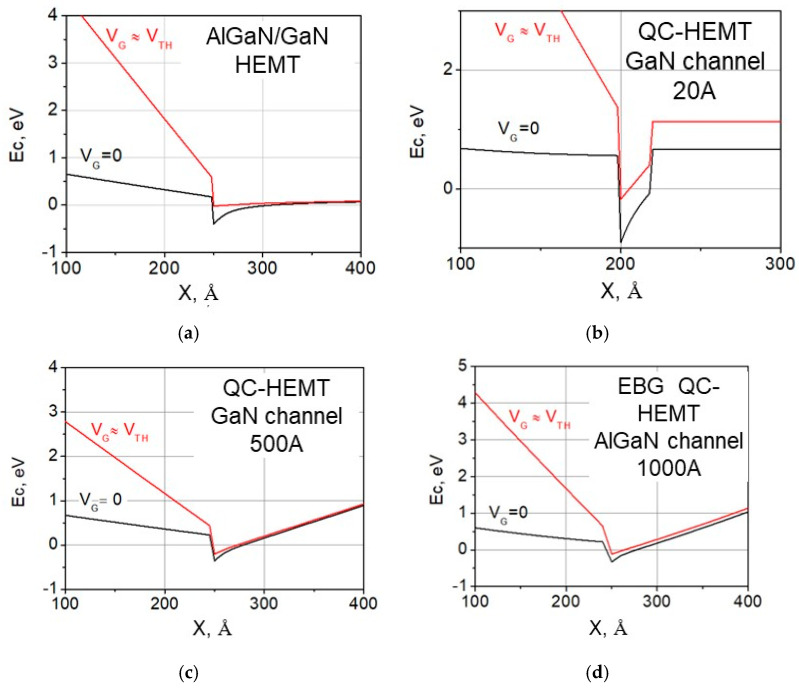
Conduction energy band diagrams of conventional HEMT (**a**) and QC-HEMTs (**b**,**c**) with different channel-barrier configurations, as shown in Table 1 below. VG and VTTH are the gate and threshold voltages correspondingly. The EBG HEMT in (**d**) is included for comparison with experimental data discussed later in this paper.

Figure 2 shows the energy band diagram of conventional AlGaN/GaN HEMT in comparison with QC HEMTs with different channel-barrier configurations. The band diagrams have been obtained using a 1D Poisson simulator by G. Shnider [53].

As compared to conventional HEMT Figure 2a, QC-HEMT design Figure 2b–d leads to two important results: first, the two-dimensional electron gas in the device channel is confined, even at gate voltages close to the threshold; and second, the ground state in the quantum well remains above the bottom of the conduction band in GaN.

As shown in Figure 2, in conventional HEMT, the band diagram flattens out at the gate voltage V_G_ close to the threshold voltage V_TH_, meaning electrons are not confined in the vicinity of the barrier–channel interface. On the contrary, in QC-HEMT the band diagram maintains its profile across the entire V_G_ range from 0 V to V_TH_. Note that the triangular shape of E_C_ profile is maintained over a broad range of QC-HEMT channel thicknesses, from 2 nm to 100 nm, as shown in the device examples in Figure 2. This is due to the fact that the E_C_ profile is mainly determined by not only by conduction band discontinuity but also by the electric field resulting from polarization charges at top and back barrier–channel interfaces. As long as the barrier and channel materials are not relaxed, this field maintains the QC-HEMT E_C_ profile.

**Figure 3:**
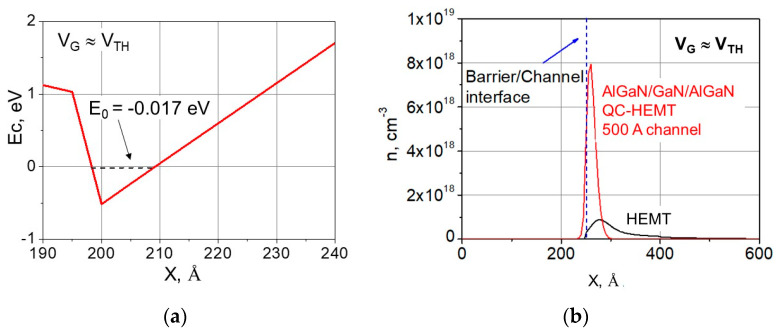
Conduction band Ec profile (**a**) and electron density profile (**b**) at V_G_ ≈ V_TH_ for the QC-HEMT with a 500 Å thick GaN channel between the 25%-Al top and back barriers. For comparison, (**b**) also shows the electron density profile for the conventional HEMT at V_G_ ≈ V_TH_.

It is important to point out that, as long as the channel material is not relaxed, the conduction band profile maintains its triangular shape. The electrons in the device channel are confined close to the bottom of the triangular potential well, and the effective thickness of the channel is determined by the QW thickness at the ground energy state *E*_0_, and not by the total channel thickness. This is illustrated in the example conduction band profile for the 50 nm QC-HEMT in Figure 3a. At a gate voltage close to threshold, the ground state energy level *E*_0_ ≈ −0.017 eV below the Fermi level. At this level, the effective width of the QW is around 1 nm. As we show below, in our experimental QC-HEMT with a 100 nm thick channel, the relaxation level is as low as 3.9%.

Figure 3b illustrates the better electron confinement in the QC-HEMT in comparison with a conventional HEMT. For this comparison, we used an example QC-HEMT with a 500 Å thick channel. This channel thickness is much higher than the characteristic width of the 2DEG profile (around 30 Å). As described above, the confined shape of the 2DEG is due to the strong polarization field in the channel.

The ground state energy *E*_0_ can be estimated as [54]

(1) $$E_{o}={\left(\frac{\hbar^{2}}{2m}\right)}^{1/3}{\left(\frac{3\pi }{2}qF_{eff}\right)}^{2/3}{\left(n_{q}+3/4\right)}^{2/3}$$

where *n_q_* is the quantum number (*n_q_* = 0 for the ground state), and *F_eff_* is the effective electric field in the channel. Our simulations show that, just like for Si MOSFETs [55], the effective electric field in the conventional HEMT is approximately

(2) $$F_{eff}\approx \frac{F_{i}}{2}=\frac{qn_{s}}{2{\epsilon \varepsilon }_{o}},$$

where *F_i_* is the electric field at the barrier-channel interface, *n_s_* is the sheet electron density in the channel, *ε_o_* is the dielectric permittivity of vacuum, and *ε* = 8.9 is the GaN dielectric constant. Factor 2 in the denominator accounts for the band bending. For the QC-HEMT $F_{eff}\approx F_{s}+\frac{F_{i}}{2}$ where *F_s_* is the polarization field that depends on the molar fractions of the cladding layers and the QC thickness. We used a self-consistent solution of the Schrödinger—Poisson equation to estimate *F_s_* = 10^8^ V/m. We also found the ground energy states for the QC-HEMT using a 1D Poisson simulator by G. Snider [53] and obtained very close results (see Table 1).

**Figure 4:**
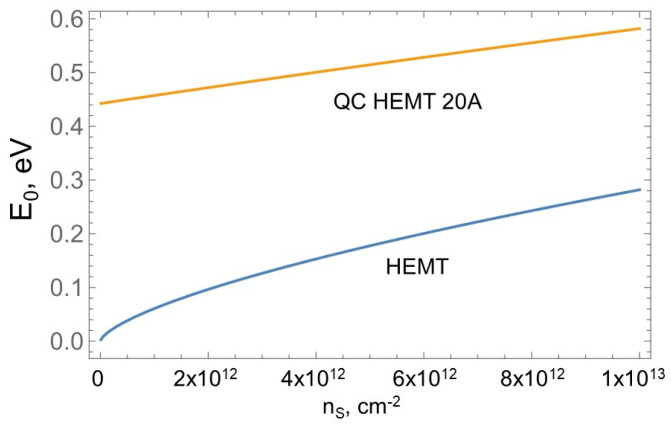
Dependencies of the ground state energy *E*_0_ above the bottom of the conduction band on the sheet carrier concentration *n_S_* for the conventional HEMT and the QC-HEMT with a 2 nm thick channel.

Figure 4 compares the dependencies of the ground state energy *E*_0_ above the bottom of the conduction band on the sheet carrier concentration *n_s_* for the conventional HEMT (Figure 2a) and for the QC-HEMT with a 20 Å thick channel (Figure 2b) generated using Equations (1)–(3).

As observed, in the conventional HEMT, at a gate voltage close to the threshold (*n_S_* => 0), the ground state energy practically coincides with the bottom of the conduction band *E_c_*. In contrast, in the QC-HEMT, the ground state energy remains well above *E_c_* in a broad range of 2DEG densities. This QC-HEMT feature makes an important impact on the device breakdown field.

*A.*  *Breakdown field in QC-HEMT*

As observed, the position of the lowest quantum state *E*_0_ in the quantum channel device remains practically constant, even at *n_s_* at the gate voltage close to the threshold. This is equivalent to the effective increase in the energy gap by *E*_0_. Note that for the breakdown field consideration, the device parameters at the gate voltage close to the threshold are particularly important. The analysis presented in [56] shows that the breakdown field, *F_CR_*, is approximately proportional to *E_G_*^2.5^, consistent with the experimental data presented in [57]. For QC-HEMT, the effective energy gap *E_Geff_* = *E_G_* + *E*_0_. For the QC-HEMT example of Figure 2b, *E_G_* = 3.39 eV, *E*_0_ = 0.45 eV at the gate voltage close to the threshold, and the resulting increase in the *F_BR_* is around 36%.

The power dependence of F_CR_ on *E_G_* can be understood by considering the ionization energy an ionizing electron must acquire $F_{CR}\lambda =\alpha E_{G}$ to generate an electron-hole pair (α > 1). Its mean free path $\lambda =v_{th}\tau \sim \tau /m^{1/2}$. Here $v_{th}\sim 1/m^{1/2}$ is the thermal velocity, *m* is the effective mass (inversely proportional to *E_G_* according to the Kane model). The scattering time τ is inversely proportional to the optical phonon energy, which, in turn, is proportional to *E_G_* resulting in $F_{CR}\sim E_{G}^{2.5}$.

Strong electron confinement in QC-HEMT may lead to another important effect resulting in further *F_BR_* increase. As shown in [58], at high electron energies, real space transfer of hot electrons from the quantum well into the barrier should occur. Electron distribution in the QC-HEMT in Figure 3 shows that strong electron confinement leads to a significant fraction of electrons penetrating the top barrier.

Therefore, it is reasonable to expect that, at a high electric field, a large fraction of the channel would experience quantum transfer to the top barrier. Since the top barrier is made of material with a larger bandgap (in our example, it is AlGaN with 65% Al), a further significant increase in the effective breakdown field *F_BR_* is expected. This effect requires further theoretical and experimental studies.

*B.*  *Electron mobility in QC-HEMT*

Strong electric confinement in the QC-HEMT leads to a smaller 2DEG effective Δ*d* thickness than in a conventional HEMT. The Δ*d* could be estimated as the ratio of the ground state energy over the electric field at the heterointerface:

(3) $$\Delta d\approx \frac{E_{0}}{qF_{i}}$$

**Figure 5:**
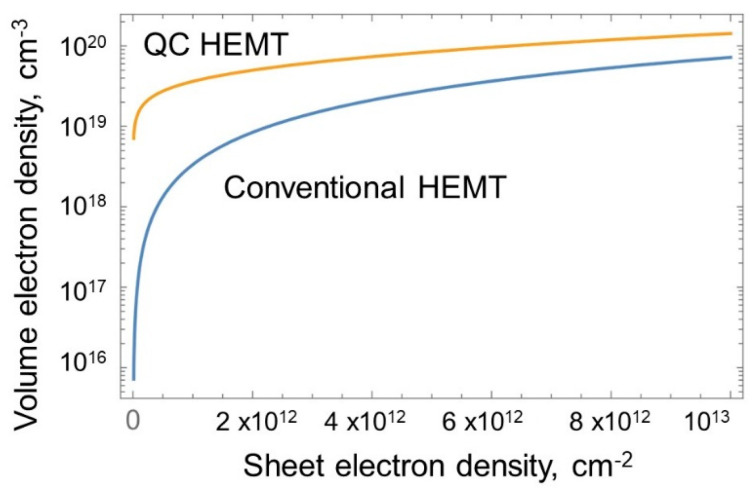
The 2DEG volume electron density as a function of n_s_ in conventional and QC HEMTs.

Hence, bulk (volume) electron density for the same *n_s_* value is higher in the QC-HEMT as compared to the conventional HEMT. Figure 5 shows the volume electron density as a function of *n_s_* in the conventional and QC-HEMT calculated using Equations (1)–(3).

**Figure 6:**
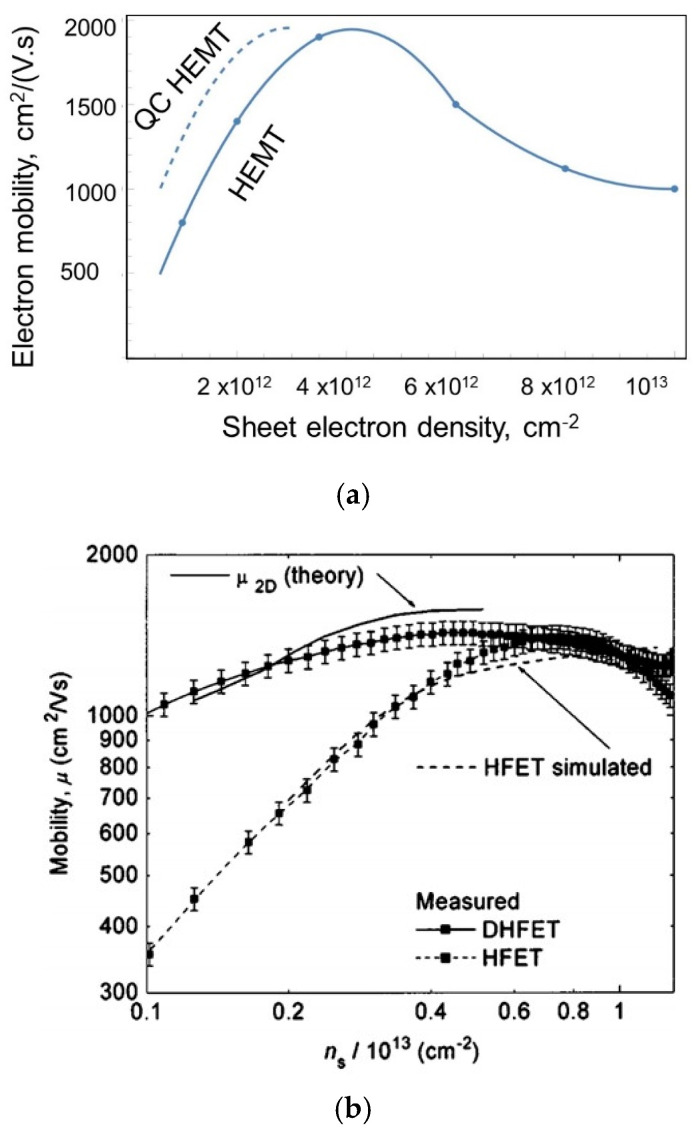
(**a**) Expected mobility increase in a QC HEMT; (**b**) experimentally observed mobility increase in double heterostructure (DH) HEMT.

As observed, in a conventional HEMT, volume electron density rapidly decreases as *n_s_* decreases, i.e., as the gate bias approaches the threshold, because the effective width of the 2DEG Δ*d* in a conventional HEMT rapidly increases as the gate bias approaches the threshold, causing 2DEG confinement to nearly disappear. In the QC-HEMT, the volume electron density is a much slower function of *n_s_*. A relative increase in volume density leads to better screening and less impurity scattering, and hence higher mobility. Figure 6a illustrates this expected improvement extracted from the measured data [28]. Experimental confirmation of increased mobility due to better confinement in double heterostructure (DH) HEMTs has been previously obtained in [59];the data are shown in Figure 6b. Field-effect mobility in III-Nitride HEMTs depends on many factors, such as interface roughness, defect concentration, strain, alloy scattering, and dislocation density. However, strong scattering screening due to higher concentration should result in higher mobility in any HEMT. A QC-HEMT with a 2 nm thick channel exhibits nearly ten times the electron confinement seen in the DH HEMT reported in [59].

In thin channel devices with tight 2DEG distribution, there are also mechanisms that lower electron mobility, such as increased phonon scattering [60] and alloy disorder scattering [61].

## 3. Experimental Validation of QC-HEMT Breakdown Field Enhancement

### 3.1. Material Growth and Device Fabrication

**Figure 7:**
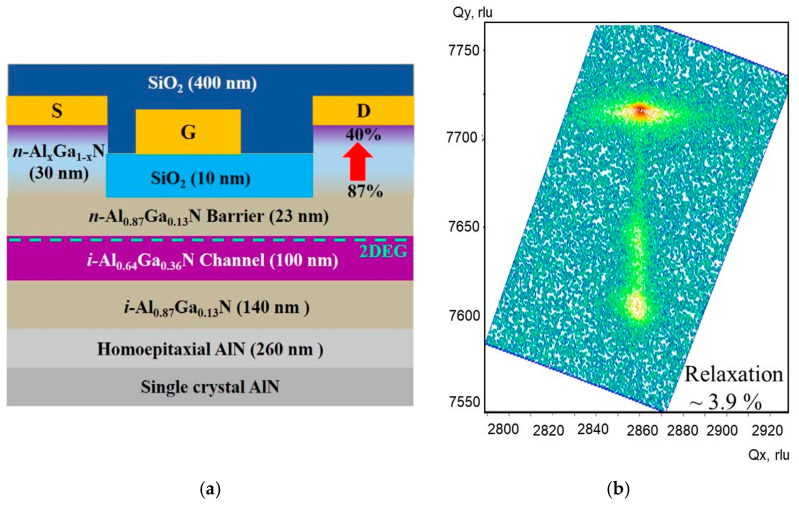
Schematic cross-section (**a**) and reciprocal space mapping of the epilayer structure (**b**) of Al_0.64_Ga_0.36_N channel insulated-gate *E_BG_* HEMT [38,39].

Pseudomorphic Al_0.87_Ga_0.13_N/Al_0.64_Ga_0.36_N/Al_0.87_Ga_0.13_N HEMTs with SiO_2_ gate insulator were grown on a single crystal bulk AlN substrate low-pressure metalorganic-chemical vapor deposition (LP-MOCVD) [38]. The 2 × 2 μm^2^ AFM scan of the AlN substrate shows uniform parallel steps with root mean square (RMS) roughness ~0.089 nm. The LP-MOCVD growth was carried out at 1100 °C and 40 torr using trimethylaluminum (TMAl), trimethylgallium (TMGa), and ammonia (NH3) as the precursors. The epilayer structure and device design are shown in Figure 7. The structure consisted of a 260 nm epitaxial AlN layer followed by a 140 nm Al_0.87_Ga_0.13_N back-barrier, a 100 nm Al_0.64_Ga_0.36_N channel layer, and a 23 nm thick Al_0.87_Ga_0.13_N barrier layer. To facilitate ohmic contact formation, the structure was capped with a 30 nm thick highly Si-doped reverse-graded Al_x_Ga_1−x_N (x = 0.87 → 0.40) layer. The reciprocal space mapping of (105) reflection shows the epilayer relaxation of 3.9% (Figure 7b). More details can be found in [38]. Note that a low relaxation level in the material supports the assumption of the triangular conduction band profile in the QC-HEMT.

The device fabrication began with mesa isolation using inductively coupled plasma reactive ion etching (ICP-RIE). A Zr/Al/Mo/Au metal stack was deposited by electron beam evaporation for the source/drain contacts, followed by rapid thermal annealing at 950 °C for 30 s. Then, ICP-RIE etching was used to remove the reverse-graded layer from the access region, and 10 nm SiO_2_ was deposited as the gate oxide. Next, the gate and probe metal stacks were deposited, consisting of Ni/Au (100/200 nm) and Ti/Ni/Au (500/700/1500 Å), respectively. Finally, the devices were capped with a 400 nm thick SiO_2_ film to prevent surface flashover. The fabricated devices had a gate length *L_G_* = 1.5 μm, a source-gate spacing *L_GS_* = 0.65 μm, various gate-drain spacings, and a width *W* = 50 μm

### 3.2. Electrical Characterization

The average on-wafer 2DEG sheet resistance measured using the Lehighton rf-mapping system was *R_SH_* = 2400 Ohm/sq. Using the transfer length method (TLM), we obtained contact resistance *R_C_* = 4.3 Ohm.mm and *R_SH_* = 2400 Ohm/sq, in close agreement with on-wafer mapping. Electron mobility of 130 cm^2^V^−1^s^−1^ at zero gate bias was extracted from C-V and transconductance measurements following the procedure described in [28]. On-resistance extracted from the device I-V was found to be 25 Ω.mm at V_G_ = +2 V.

**Figure 8:**
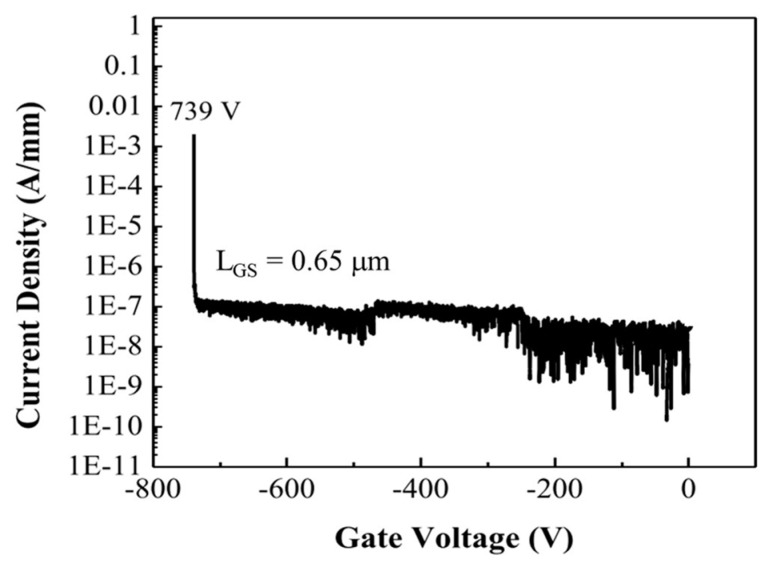
EBG HEMT breakdown current-voltage characteristics [1].

**Figure 9:**
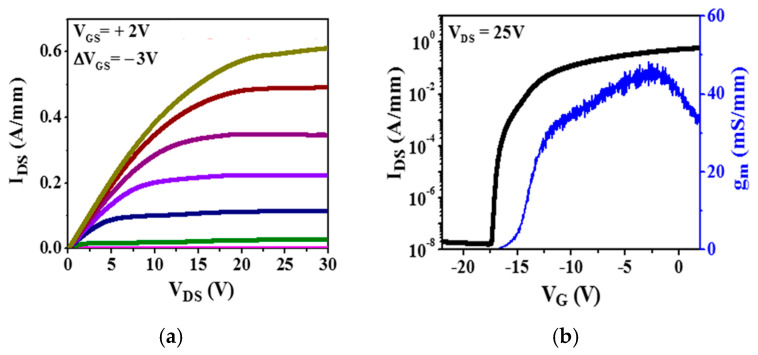
EBG HEMT drain current I_D_ (V_D_) (**a**), transfer ID (VG), and transconductance g_m_ (V_G_) (**b**) characteristics [39].

We then measured a two-terminal breakdown voltage between the gate and source electrodes with *L_GS_* = 0.65 μm spacing. Electrode spacing was verified using SEM imaging. We kept short electrode spacing to reduce electric field non-uniformity and prevent surface flashover. Figure 8 shows the current-voltage characteristics for breakdown measurements. From the measured breakdown voltage *V_BD_* = 739 V, the average electric field at breakdown *F_BD_* = 11.37 MV/cm was found. Drain current and transconductance I-V characteristics for EBG HEMT are shown in Figure 9 [39].

## 4. Discussion of the Experimental Results

The measured FBD value presents a low bound for the actual breakdown field value as the field distribution is non-uniform. Therefore, the breakdown occurs in the electric field peak that forms at the contact metal edge. Using Synopsys, our 2D simulations of the electric field profiles showed that the peak field is at least 15% higher than the average field (reaching approximately 13.1 MV/cm). This value is close to that estimated for the Al_0.87_GaN_0.13_N barrier material, and, as shown below, is expected for a QC-HEMT design.

The breakdown field of the HEMT device can be estimated based on the composition of the device channel material. Using Vegard’s law with the bandgaps of GaN *E_G_*_1_ = 3.4 eV and AlN *E_G_*_2_ = 6.2 eV and bowing factor *b* = 0.7 [62], we obtain fraction *x* = 0.64 for the channel Al

*E_GCH_* = *xE_G_*_2_ + *E_G_*_1_ (1 − *x*) − *bx*(1 − *x*) = 5.03 eV, (4)

Next, using the bandgap–critical field relationship after [56]

*F_CR_* = 0.173 *E_G_*^2.5^, (5)

we obtain *F_CR_* = 9.8 MV/cm. As observed, the expected critical field is lower than the measured value. To explain this breakdown field increase, we involve the quantization effects in quantum channel devices. In QC-HEMT, the channel region forms a quantum well (QW), whose profile is mainly determined by the polarization charges at the top and bottom interfaces. Due to this, the QW maintains the triangular shape and, hence, supports strong confinement even at the gate bias below the threshold. In conventional HEMTs, on the contrary, the E_C_ profile completely flattens out at gate bias close to V_TH_. The band diagrams of a EBG QC-HEMT and a conventional HEMT at different gate biases are compared in Figure 2.

The average electric field in the QC-HEMT channel estimated from the slope of the band diagram is *F_CH_* ≈ 0.5 MV/cm. For an infinitely high triangular barrier, the lowest state energy is given by [63]:

(6) $$E_{o}=c_{1}\;{\left[\frac{(qF_{CH}\hbar {)}^{2}}{2m_{EF}}\right]}^{\frac{1}{3}\;}$$

where *c*_1_ = 2.338, and *m_EF_* is the effective electron mass in the channel. Using linear interpolation between GaN and AlN, we found *m_EF_* = 0.34 *m*_0_ for our 64%-Al AlGaN channel. The lowest energy level given by (6) is *E*_0_ = 0.16 eV above the conductance band edge EC.

**Figure 10:**
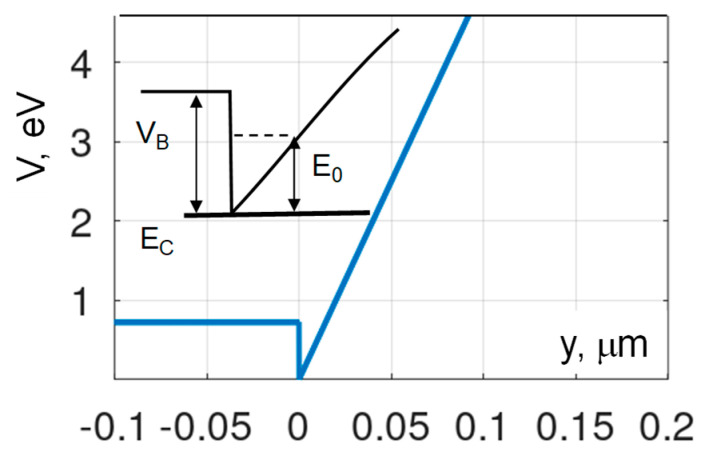
Triangular QW profile used in MATLAB calculations of the ground state energy *E*_1_. The barrier height at the Al_0.87_Ga_0.13_N/Al_0.64_Ga_0.36_N barrier/channel interface, *V_B_* = 0.72 eV, electric field in the channel *F_CH_* = 0.5 MV/cm, electron effective mass in the channel, *m_EF_* = 0.34 *m*_0_.

The barrier/channel height calculated for our EBG HEMT composition is *V_B_* = 0.72 eV. Using MATLAB to find eigenvalues of the Schrödinger equation for a triangular QW with finite barrier height (see Figure 10) yielded a more accurate value of *E*_0_ = 0.13 eV.

As illustrated in Figure 10, the quantization in the channel results in a larger effective bandgap *E_GEF_* than that corresponding to the material composition: *E_GEF_* = *E_G_* + *E*_0_ = 5.16 eV (here we ignored quantization in the valence band). A higher *E_GEF_* translates into a higher critical breakdown field evaluated as [56].

*F_CR_*_1_ = 0.173*E_GEF_*^2.5^ = 10.46 MV/cm (7)

The critical field accounting for the quantization effects in the QC-HEMT is closer to the measured value of 11.37 MV/cm, but is still lower. Therefore, the quantization of energy states in the QC-HEMT explains only a fraction of the excessive critical field observed in the experiment.

An additional important mechanism enhancing the breakdown field is the effect that we call the electron quantum real space transfer into the Al_0.87_Ga_0.13_ barrier layer. As shown in [58], a larger density of states in quantum well cladding layers causes the wave function penetration into the cladding layers to increase with an increase in the electron kinetic energy *E_kin_*, where *k* is the electron momentum in the channel plane. This transfer reduces the effective barrier height and completely eliminates the effective barrier for energetic electrons capable of causing impact ionization in the channel. The fraction of the remaining barrier height *f_b_* is a function of the channel electrons kinetic energy *E_kin_* to the barrier height *V_B_* ratio [28]:

(8) $$f_{B}=\sqrt{1-E_{0}/V_{B}-E_{kin}/V_{B}\;\left(m_{B}/m_{EF}-1\right)}$$

**Figure 11:**
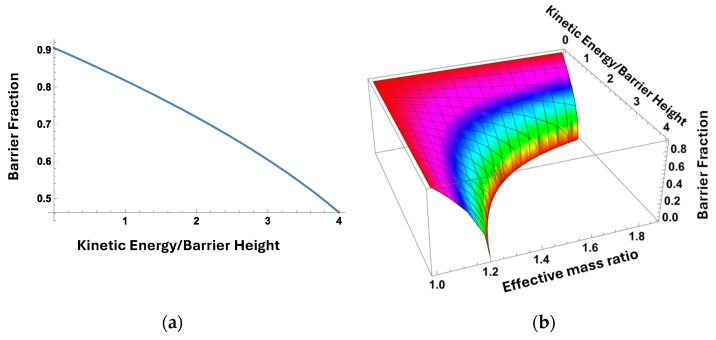
Barrier reduction for energetic electrons: (**a**) effective barrier height versus kinetic energy of electrons for effective mass ratio in the QC-HEMT studied in this paper and (**b**) effective barrier height versus kinetic energy of electrons for all mass ratios for AlN/GaN system.

Here, *m_B_* and *m_EFF_* are the electron effective masses in the barrier and channel layers. In Figure 11a, we plotted the effective barrier reduction for the device parameters of our QC HEMT. As seen from the figure, the effective barrier disappears for electrons with $E_{kin}/V_{B}\;\geq 4$. Electrons causing impact ionization have kinetic energy *E_kin_* > 6.2 eV or $E_{kin}/V_{B}>8$. Therefore, all very energetic electrons responsible for ionization should transfer into the cladding layer and the expected breakdown field should be equal to the breakdown field in the cladding layer, in agreement with the measured breakdown field.

Figure 11b shows the barrier reduction as a function of both e1 and. The upper bound of the range of 1.87 corresponds to the effective mass ratio of AlN and GaN. As seen from the figure, a large difference in the Al molar fraction between the barrier and the channel is beneficial for the quantum real space transfer. Such designs have the additional advantage of having much higher electron mobility in the device channel, which can be achieved by making the channel thinner to avoid the development of dislocation arrays.

It is interesting to compare the obtained breakdown field in the EBG QC-HEMT device with other data available for conventional HEMTs and double heterostructure HEMTs with epilayer structures similar to that of the QC-HEMT. Numerous results have been published on breakdown in single heterostructure GaN- and AlGaN-channel HEMTs, with the vast majority of them reporting the breakdown fields considerably lower than those expected from material parameters. One of the highest breakdown fields, 1.6 MV/cm, have been achieved in Al_0.15_Ga_0.75_N HEMT [64]. As pointed out in [65], the breakdown fields achieved in double heterostructure HEMTs are typically significantly (2–3 times) higher than those in single heterostructure devices. This observation is in agreement with the concept of QC-HEMT.

## 5. Conclusions

In conclusion, the QC Extreme Bandgap AlGaN HEMT design demonstrated the potential to considerably improve the breakdown voltage. QC-HEMT approach also has strong potential to increase field-effect mobility, and therefore transconductance, at low electron sheet densities, while supporting a large maximum electron sheet density and a larger maximum current. A thinner quantum well made of lower bandgap material should also lead to better radiation hardness in QC-HEMT. 

## Figures and Tables

**Table 1:** Details of device structures shown in Figure 2. The position of ground state energy *E*_0_ above conduction band edge E_C_ was found using Equation (2) below and from 1D Poisson simulations [53].

	Conventional HEMT	Thin Channel QC HEMT	AlGaN/GaN/AlGaN QC-HEMT	EBG AlGaN QC-HEMT
Channel material	GaN	GaN	GaN	Al_065_Ga_0.35_N
Channel thickness	-	20 Å	500 Å	1000 Å
Al fraction in top/back barriers	25%	25%/25%	25%/25%	87%/87%
Ground state *E*_0_ location above E_C_ at V_G_ ≈ V_TH_	-	0.54 eV	0.42 eV	0.15 eV

## Data Availability

Data are contained within the article.
